# Some Recent Advances in Paediatrics
*From an address delivered to the Torquay & District Medical Society on Thursday, March 9th, 1950.


**Published:** 1950-04

**Authors:** Wilfrid P. H. Sheldon

**Affiliations:** Physician to Out-Patients, The Hospital for Sick Children, Great Ormond Street; Physician-in-Charge, Children's Department, King's College Hospital, etc.


### SOME RECENT ADVANCES IN PAEDIATRICS*

BY

WILFRID P. H. SHELDON, M.D., F.R.C.P.

*Physician to Out-Patients, The Hospital for Sick Children, Great Ormond Street; Physician-in-Charge, Children's Department, King's College Hospital, etc.*

---

The first condition I want to mention is concerned with " milk dryness ". This occurs in primiparae who at the commencement of lactation seem to have an adequate supply of milk which dries up after two or three weeks. It has been found that this is due to the fact that the baby does not empty the breast completely at each meal, so that a residue of milk is left: and this, accumulating and causing pressure in the breast acini, brings about the cessation of milk secretion. This condition can be overcome by teaching the mothers to empty the breasts themselves by manual manipulation after feeding. At the British Hospital for Mothers and Babies at Woolwich this excess milk forms the nucleus of the milk bank which has proved so useful in supplying human milk when required for premature babies both at this and other hospitals.

Next let us turn our attention to the importance of neonatal sepsis. It has to be remembered that the new-born has not had time to attain any real resistance to infection. Even the mildest degree of infection such as a sticky umbilicus or gummy eyes is likely to prove dangerous and may well lead to septicaemia. The picture of sepsis in the new-born is quite unlike that in the adult: the condition should be suspected if the child vomits after its feeds, shows a low and diminishing temperature and ultimately goes off its feeds and starts having convulsions. The cause of these symptoms is often mistaken for a digestive condition rather than infection and so the diet is modified in various ways. In actual fact however the trouble is probably septic rather than

* From an address delivered to the Torquay & District Medical Society on Thursday, March 9th, 1950.

dietetic. The treatment in this case is to give penicillin at once in adequate doses—up to 10,000 units per pound—until the infant recovers. This dosage could be given orally but is more efficient when given by injection.

The next condition to be considered is pyloric stenosis. Here it is essential to withhold eumydrin until the diagnosis has been quite definitely established. Another cause of vomiting in infants is a condition which has only recently been recognized and is known as lax cardia. This is due to a developmental error resulting in a " short oesophagus " so that there is no sphincter reaction at the cardia. Consequently, when the infant is put down after feeding the meal regurgitates up the oesophagus. As a result ulceration and erosion of the oesophagus will take place and after a time the vomit will contain specks of blood, which will distinguish the condition from vomiting due to pyloric stenosis. The treatment for this condition is to keep the infant in an upright posture after every meal and to give it alkalis at the conclusion of the feed.

Vomiting and choking which take place during feeding may be due to immaturity of the neuro-muscular mechanism of the pharynx and larynx. This is a condition occurring in the very young and must be distinguished from vomiting and choking due to atresia of the oesophagus. Examination under the X-ray screen following administration of a teaspoonful of opaque oil will settle the diagnosis. It may be necessary to feed the child by means of a tube until the reflexes of the pharynx are fully established.

*Steatorrhoea.* One of the more recently recognized causes for this lesion is fibrocystic disease of the pancreas. It is a familial condition and is the result of obstruction of the pancreatic ducts by viscid mucus. The pancreatic secretion is thus damned back, so that the meconium is not digested and becomes almost rubber-like, leading to meconium ileus and obstruction. At this latter stage the condition is a surgical problem. Children affected with this condition are difficult to nourish and do not put on weight. Their stools are most offensive; they are particularly prone to suppurative infections of the chest. The familial aspect of the disease is often evident from the mother's story; she may have had one baby which died from intestinal obstruction and others from pneumonia. If the condition is suspected it can be confirmed by passing a duodenal tube under X-ray control and withdrawing duodenal secretion: it will be found in such cases that pancreatic trypsin is absent. Coeliac disease is another form of steatorrhoea : this disease differs from the last type of steatorrhoea in that it usually comes on between the ages of six months and two years. It is probably not due primarily to inability to digest fat (as was previously supposed) but to inability to cope with starch, and the distention found in this disease is due to starch fermentation. It has now been established that if children are put on a starch-free diet (soya-meal biscuits are of great assistance) and given up to 50 grammes of fat a day they improve very rapidly and after a time become normal.

*Hirschsprung's Disease.* It has now been found that in this condition the hypertrophic colon is in fact secondary to the rectum being unable to receive the contents of the colon and it is the rectum which is at fault. Treatment is surgical. After a colostomy, the rectum is excised leaving the anal sphincter, and the pelvic colon is brought down and attached to the inner margin of the anus. These cases do well with this procedure.

About B.C.G. inoculation: it takes an infant six to ten weeks to develop resistance as a result of inoculation : and meanwhile any risk of the child being infected with the tubercle bacillus must be guarded against. Therefore it is advisable that the child should be kept in a tubercle-free atmosphere for a period of at least six weeks both before it is inoculated and afterwards.

